# Automatic Determination of the Center of Macular Hole Using Optical Coherence Tomography En Face Images

**DOI:** 10.3390/jcm11113167

**Published:** 2022-06-02

**Authors:** Takanori Sasaki, Takuhei Shoji, Junji Kanno, Hirokazu Ishii, Yuji Yoshikawa, Hisashi Ibuki, Kei Shinoda

**Affiliations:** Department of Ophthalmology, Saitama Medical University, Saitama 350-0495, Japan; shoojii@gmail.com (T.S.); kannojunichi55@gmail.com (J.K.); number21garnett@yahoo.co.jp (H.I.); yuji.yoshi.md@gmail.com (Y.Y.); ceupawiri@gmail.com (H.I.); shinodak@med.teikyo-u.ac.jp (K.S.)

**Keywords:** idiopathic macular hole, optical coherence tomography, automation, ImageJ

## Abstract

To evaluate the automated determination of the center of an idiopathic macular hole (MH) by using swept-source optical coherence tomography (OCT) images with new macro-based algorithms in ImageJ and to compare the difference between the MH center measurements obtained automatically and manually. This cross-sectional study included 39 eyes of 39 elderly individuals (22 women, 17 men) with stage 3 and 4 MH. The MH center was automatically determined using the ImageJ macro. The foveal center was also manually identified by two masked examiners using horizontal and vertical serial B-scan OCT angiography images. The mean age was 68.8 ± 8.3 years. After adjusting for the effect of magnification, the mean distance between the MH center determined manually by Examiner 1 and that determined automatically was 15.5 ± 9.9 µm. The mean distance between the two manually determined measurements of the MH center was 20.3 ± 19.7 µm. These two mean distance values did not differ significantly (Welch *t*-test, *p* = 0.27) and was non-inferior (*p* < 0.0001). The automated ImageJ-based method for determining the MH center was comparable to manual methods. This study showed that automated measurements were non-inferior to manual measurements, and demonstrated a substitutable usefulness, at least for use in clinical practice.

## 1. Introduction

An idiopathic macular hole (MH) is a common macular disease [1] that involves tissue defects in the fovea, including the photoreceptor layer. Optical coherence tomography (OCT) is a noninvasive retinal cross-sectional imaging technique [2] that can be used to easily measure the structure of a MH. The center of the fovea is the area with the highest visual acuity and the highest density of cone photoreceptors; thus, an MH causes decreased vision, metamorphopsia, and a central dark spot [3]. MH can be closed and treated via pars plana vitrectomy (PPV) [4], and the closure rate can be significantly increased by peeling the internal limiting membrane (ILM) during PPV [5]. Other surgical techniques, such as the use of an ILM inverted flap within the MH [6], amniotic membrane patch [7], and autologous retinal transplant [8], have also been developed and reported.

Anatomical migration after MH surgery was reported recently [9,10,11,12,13,14,15]. Fundus photography and OCT revealed that the fovea was displaced to the optic disc following MH closure via vitrectomy with ILM peeling. The center of the MH is a landmark for measuring the movement of the retina after surgery; therefore, it is crucial to identify it with high accuracy and reproducibility. However, at present, the center of the MH is mostly identified manually [9,10,11,12,13]. Therefore, we investigated whether it is feasible to determine MH center using an automated image analysis technique. En face OCT can assess retinal structures and can obtain images at different retinal depths [16]. In some previous reports, the extent of the foveal avascular zone (FAZ) was obtained by automated measurement using en face OCT [17,18]. The MH presents as a hypo-reflective cavity surrounded by the high-contrast boundaries of the retina. These clean-cut boundaries are easy to analyze using a computer. Several automatic measurement methods using en face OCT images have been reported recently. Ishii et al. reported an automatic method for measuring the FAZ [19], and Shoji et al. reported an automatic method for measuring the center of the FAZ [20]. Philippakis used ImageJ software to measure MH size, and this software can also be used to detect MH center [21]. However, information regarding the measurement of MH center, which we believe is clinically relevant in investigating MH movement after its closure, is currently not available. Thus, the aim of this study was to propose a feasible and reliable automated method for detecting MH center using en face OCT scans. We also sought to compare automated and manual detection methods.

## 2. Materials and Methods

### 2.1. Study Population

This retrospective observational case series was conducted in accordance with the tenets of the Declaration of Helsinki. The retrospective review of patient records was approved by the Ethics Committee of Saitama Medical University (IRB 19079.03). Consent was obtained from all patients. Among patients who visited Saitama Medical University Hospital between 1 February 2018 and 30 November 2019, who showed MH and underwent preoperative OCT imaging, 42 patients (25 men and 17 women) with stage 3 and 4 MH defined by the Gass [22] classification were included in this study. The exclusion criteria were as follows: (1) patient age < 20 years and (2) poor image quality (signal strength < 8 due to signal noise; 1 = minimum, 10 = maximum). A Carl Zeiss swept-source OCT system (PLEX^®^ Elite 9000) was used to image the macular area (3 × 3 mm) once per eye.

### 2.2. Automatic Detection

**Figure 1:**
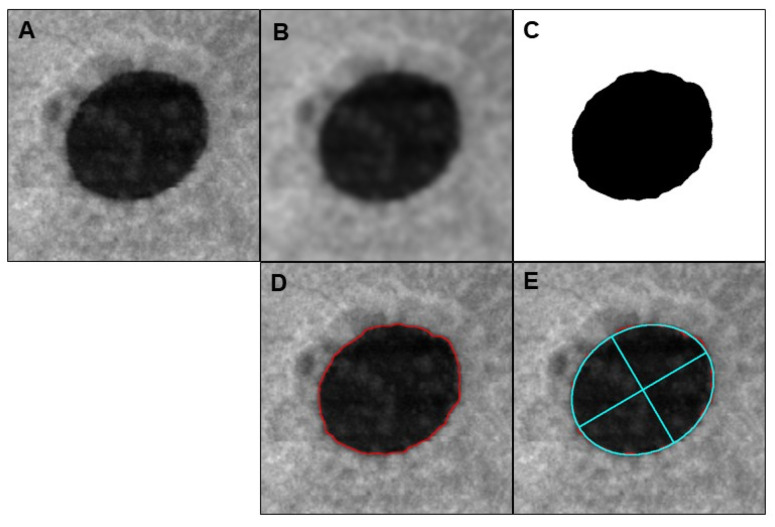
Identifying the center of the macular hole automatically in the en face images. (**A**) Original en face image (**B**) Blurring for smoothing edges (**C**) Binarization (**D**) Extraction the edge of macular hole (**E**) Centroid: detecting pore and its center of gravity.

To detect the MH center, we used an automated analysis program based on an ImageJ macro that we devised. In the introduction, we mentioned that the MH en face image is drawn as a circular area of structural defects, but these structural defects show different circles depending on the height of the retinal layer. For example, the en face image of the retinal layer at the height where the pore is the smallest and the en face image of the retinal layer at the height of the bottom of the pore have different shapes of circles; therefore, the calculated center may be different. We combined all en face images at various heights and found that the region of the structural defect in the composite image was equivalent to the area of structural defect at the height of the smallest circular hole (Supplemental Video S1). In the automatic analysis using ImageJ, we approximated the extent of the foramen defect as an ellipse and determined its center of gravity, which we used as the center of the MH. The program is described in detail below (Algorithm 1).
**Algorithm 1**. ImageJ Macrorun(“Median...”, “radius = 2”);run(“Gaussian Blur...”, “sigma = 5”);run(“Auto Local Threshold...,” “method = Phansalkar radius = 15 parameter_1 = 0 parameter_2 = 0 white”).doWand(512, 512);roiManager(“Add”);

The image-processing algorithm is illustrated in Figure 1.

### 2.3. Manual Detection

**Figure 2:**
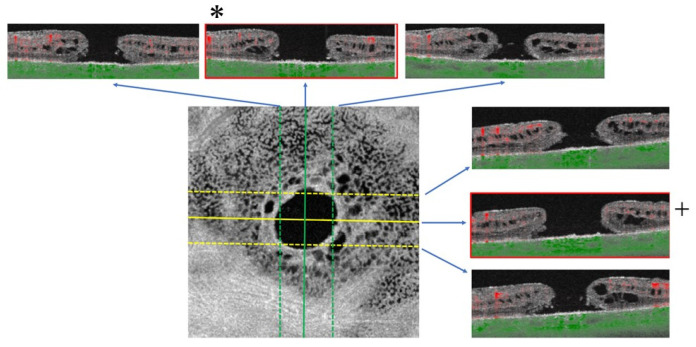
Identifying the center of the macular hole in an en face image manually. Examiner checked all horizontal and vertical scan image, and detected longest hole diameter images (horizontal; +, vertical; *). The center of macular hole is the intersection of the slices of the area with the largest diameter in the horizontal and vertical B-scan images of the OCT. The center point was output to en face image.

In the manual method, two masked examiners (Examiner 1: T.S. (Takanori Sasaki) and Examiner 2: H.I. (Hirokazu Ishii)) independently detected the MH center using B scan images. The examiners used the scale parameter of the software, which was set to define a 1024-pixel width in the images as 3 mm. The center of the MH was defined as the point of intersection between the slices of the area with the largest foramen diameter in the horizontal and vertical B-scan images. The center point was output to en face image (Figure 2, Supplemental Video S2).

### 2.4. Statistical Analysis

**Figure 3:**
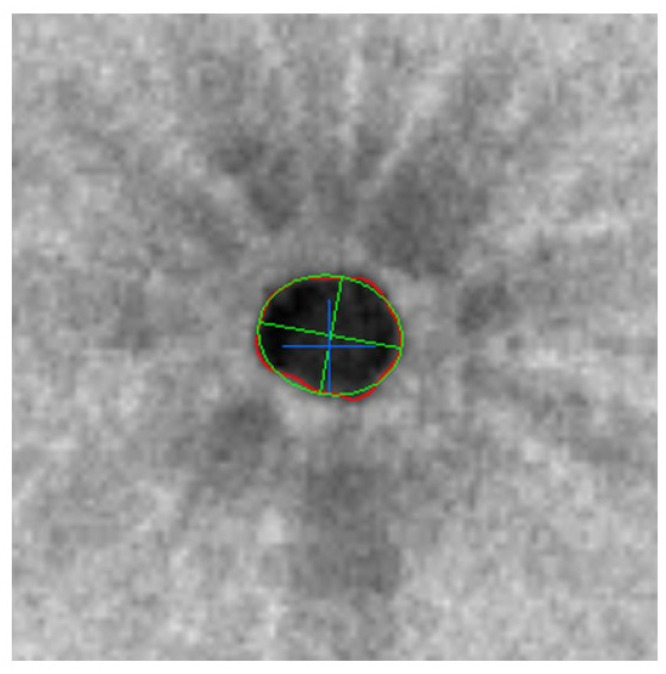
Representative case (3 × 3 mm en-face image). MH center distance between manual and auto was 22 μm. Automatic detection; Extract macular hole edge showed as red line, then detect pore center (green circle, major and minor axes). Manual detected point was shown as blue cross.

Continuous variables are expressed as mean values and standard deviations (mean ± SD). We compared the absolute value of the distance between MH center measurements obtained manually and automatically (Examiner 1—Automatic), as shown in Figure 3, and between MH center measurements obtained by the examiners (Examiner 1—Examiner 2). Manual methods are routinely used to identify MH center. In this study, a non-inferiority test was used to confirm the detection accuracy of MH center using the automatic method. Sample size calculation was performed. We estimated that 32 patients would be required for 95% confidence intervals and 15 μm non-inferiority margin, according to our pilot study. Welch *t*-test was used to compare continuous data. Statistical significance was set at *p* < 0.05. All statistical analyses were performed using JMP software (version 10.1; SAS Institute Inc., Cary, NC, USA).

## 3. Results

A total of 42 eyes of 42 patients with idiopathic MHs were included in this study. Automatic measurements could be obtained on the en face OCT scans of 39 of the 42 eyes. The scans for two eyes showed poor extraction because of the presence of tissue inside the pore, and one eye could not be recognized as a circle. The mean age of the 39 patients was 68.8 ± 8.3 years. Preoperative MH stages were stage 3 in 19 eyes (48.7%) and stage 4 in 20 eyes (51.3%). The mean axial length was 24.1 ± 1.9 mm. The mean MH size was 0.182 ± 0.137 mm^2^, and the mean major and minor axes were 479 ± 184 and 420 ± 174 μm, respectively (Table 1).

### Comparison of Manual and Automatic MH Center Measurements

**Figure 4:**
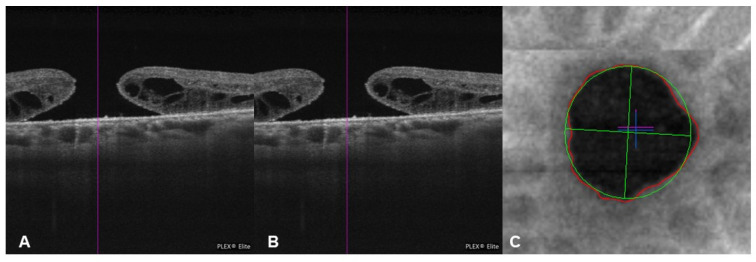
The longest macular hole center distance case between manual and automatic detection. (**A**) Vertical B scan image of macular hole center; purple line: manual detection. (**B**) Horizontal B scan image of MH center; purple line: manual detection. (**C**) The distance between the MH center measurements obtained automatically and manually was 41μm; green cross: automatic, blue and purple cross: manual.

**Figure 5:**
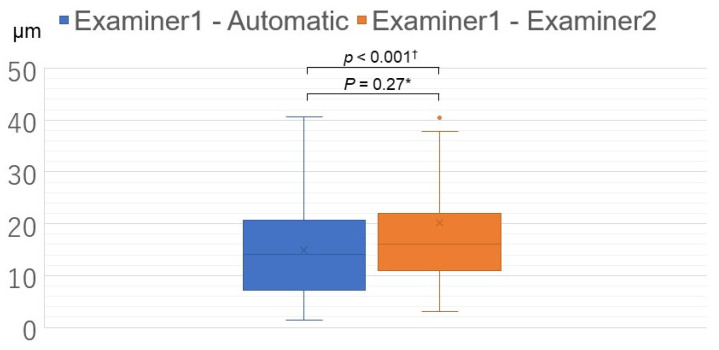
Comparison between the distance between macular hole (MH) center measurements obtained manually and automatically (Examiner 1—Automatic) and the distance between both manually obtained MH center measurements (Examiner 1—Examiner 2). * Welch *t*-test; † Non-inferiority test with the margin set at 15 μm.

The mean distance between MH center measurements obtained manually and automatically (Examiner 1—Automatic) was 15.5 ± 9.9 μm, and the longest distance was 41 μm (Figure 4). The mean distance between both manually obtained MH center measurements (Examiner 1—Examiner 2) was 20.3 ± 19.7 μm, and the longest error was 56 μm. No significant difference was observed between these mean distance values (Welch *t*-test, *p* = 0.27). Based on these results, a non-inferiority test was performed, with the non-inferiority margin set at 15 μm, and the difference between the automatic and manual measurement methods proved to be non-inferior (*p* < 0.0001). (Figure 5). In 19 eyes (48.7%), manual measurements were more nasal than automatic measurements. In 20 eyes (51.3%), automatic measurements were more nasal than manual measurements.

## 4. Discussion

In a series of 39 eyes with stages 3 and 4 MH, en face SD-OCT analysis enabled feasible and accurate automated detection of MH center. Many programs have been recently devised to automatically analyze the area, major diameter, and minor diameter of the MH [21,23], but no program has been developed to automatically extract MH center; thus, our program can be considered to be unique in this regard. We cannot prove that these errors were predominantly small, because no previous study has reported an automatic measurement method for MH center. I n a previous study, the measurement error of the automatic measurement of the macular center of normal eyes was 71 μm [24]. Therefore, we believe that the measurement error in our study is small enough.

We consider that this automatic measurement method has three advantages over the conventional manual measurement method. First, the manual measurement method requires considerable time to identify all B scan images, after checking them in both the horizontal and vertical directions. On the other hand, the automatic measurement can be performed in a few seconds with the ImageJ program. Second, since the results of manual measurement may differ from examiner to examiner, the automatic measurement method seems more useful, from the viewpoint of reproducibility. Third, the automatic analysis can help to define the center of MH, which is not necessarily a normal circle. Phillippakis et al. pointed out that the MH is not always circular, and it can be asymmetric [21]. Thus, the center of the MH, as measured by automatic analysis, may deviate from that identified by manual evaluation of B-scan images. Further, the center of the maximum diameter in the horizontal direction and that in the vertical direction do not always match.

We also calculated the distance between the MH center determined manually ((Examiner 1 + Examiner 2)/2) and the auto-detected point, and the distance was 18.2 ± 12.7 µm. Fortunately, the gap between manual and automated measurements is not large. This study confirmed that the conventional manual measurement method is also a useful measurement method.

Chen et al. [23] constructed a three-dimensional model of MH automatically and detected the zone of the minimal area manually. The authors mentioned their measurement is ‘approximately’. It is difficult to overcome the ambiguity in manual measurement, and the automated method can solve this problem.

### 4.1. Future Outlook

As mentioned above, anatomical migration after MH surgery was reported recently [9,10,11,12,13,14,15]. Although preoperative and postoperative changes in MH have been extensively studied, most of the measurement methods are manual. We believe it is possible to automatically measure the distance and direction of macular center migration before and after MH surgery.

### 4.2. Limitations

This study had several limitations. First, automatic extraction was based on the circular defect area in the en face slab. However, in MH analyses in the early stages, such as stages 1 and 2, the retinal surface layer is reflected in the area of the circular defect, and the circle cannot be drawn because it is impossible to binarize the area between the circular defect and the rest of the retinal surface layer. In this study, we could only determine the center of the full-layer MH in stages 3 and 4; therefore, future studies should aim to develop a program that can identify MH center at an early stage. Second, we analyzed the circular defect area in the en face image; therefore, this technique cannot be used for automatic extraction of the macular center after surgery, because the defect area in the en face image is considered to disappear when the MH is closed after vitrectomy. Thus, in order to automate the measurement of the movement of the macular center before and after MH surgery, it may be necessary to devise a program that can automatically measure the postoperative macular center.

## 5. Conclusions

In conclusion, our automated ImageJ-based method for determining MH center was comparable to manual methods. This study showed that automated measurements were non-inferior to manual measurements, and demonstrated a substitutable usefulness, at least for use in clinical practice.

## Figures and Tables

**Table 1:** Participants’ baseline characteristics.

Participants	
Total (*n*)	39
Age (mean ± SD, years)	68.8 ± 8.3
Sex	
Women	22
Men	17
Axial length, mm (mean ± SD)	24.1 ± 1.9
Macular hole stage (stage 3, stage 4)	(19, 20)
Macular hole size, mm^2^ (mean ± SD)	0.182 ± 0.137
Macular hole major axis, μm (mean ± SD)	479 ± 184
Macular hole minor axis, μm (mean ± SD)	420 ± 174
SD, standard deviation	

## Data Availability

The data that support the findings of this study are available from the corresponding author, T.S. (Takanori Sasaki), upon reasonable request.

## Supplementary Materials

The following supporting information can be downloaded at: https://www.mdpi.com/article/10.3390/jcm11113167/s1, Video S1: The area of the structural defect in the composite image of all en face images at various heights and at the height of the smallest circular hole. Both are equivalent; Video S2: Identifying the center of the macular hole in an en face image manually.

## References

[1] Ezra E. (2001). Idiopathic Full Thickness Macular Hole: Natural History and Pathogenesis. Br. J. Ophthalmol..

[2] Drexler W. (2004). Ultrahigh-Resolution Optical Coherence Tomography. J. Biomed. Opt..

[3] Meuer S.M., Myers C.E., Klein B.E., Swift M.K., Huang Y., Gangaputra S., Pak J.W., Danis R.P., Klein R. (2015). The Epidemiology of Vitreoretinal Interface Abnormalities as Detected by SD-OCT: The Beaver Dam Eye Study. Ophthalmology.

[4] Kelly N.E., Wendel R.T. (1991). Vitreous Surgery for Idiopathic Macular Holes: Results of a Pilot Study. Arch. Ophthalmol..

[5] Kumagai K., Furukawa M., Ogino N., Larson E., Uemura A. (2007). Long-Term Outcomes of Macular Hole Surgery with Triamcinolone Acetonide-Assisted Internal Limiting Membrane Peeling. Retina.

[6] Michalewska Z., Michalewski J., Adelman R.A., Nawrocki J. (2010). Inverted Internal Limiting Membrane Flap Technique for Large Macular Holes. Ophthalmology.

[7] Rizzo S., Caporossi T., Tartaro R., Finocchio L., Franco F., Barca F., Giansanti F., Human A. (2019). A Human Amniotic Membrane Plug to Promote Retinal Breaks Repair and Recurrent Macular Hole Closure. Retina.

[8] Grewal D.S., Mahmoud T.H. (2016). Autologous Neurosensory Retinal Free Flap for Closure of Refractory Myopic Macular Holes. JAMA Ophthalmol..

[9] Pak K.Y., Park K.H., Kim K.H., Park S.W., Byon I.S., Kim H.W., Chung I.Y., Lee J.E., Lee S.J., Lee J.E. (2017). Topographic Changes of the Macula After Closure of Idiopathic Macular Hole. Retina.

[10] Kawano K., Ito Y., Kondo M., Ishikawa K., Kachi S., Ueno S., Iguchi Y., Terasaki H. (2013). Displacement of Foveal Area Toward Optic Disc After Macular Hole Surgery with Internal Limiting Membrane Peeling. Eye.

[11] Ishida M., Ichikawa Y., Higashida R., Tsutsumi Y., Ishikawa A., Imamura Y. (2014). Retinal Displacement Toward Optic Disc After Internal Limiting Membrane Peeling for Idiopathic Macular Hole. Am. J. Ophthalmol..

[12] Ohta K., Sato A., Senda N., Fukui E. (2017). Displacement of Fovea Toward Optic Disk After Macular Hole Surgery with Internal Limiting Membrane Peeling. Int. Med. Case Rep. J..

[13] Takeyama A., Imamura Y., Fujimoto T., Iida T., Komiya Y., Shibata M., Ishida M. (2022). Retinal Displacement and Intraretinal Structural Changes After Idiopathic Macular Hole Surgery. Jpn. J. Ophthalmol..

[14] Goto K., Iwase T., Akahori T., Yamamoto K., Ra E., Terasaki H. (2019). Choroidal and Retinal Displacements After Vitrectomy with Internal Limiting Membrane Peeling in Eyes with Idiopathic Macular Hole. Sci. Rep..

[15] Akahori T., Iwase T., Yamamoto K., Ra E., Kawano K., Ito Y., Terasaki H. (2018). Macular Displacement After Vitrectomy in Eyes with Idiopathic Macular Hole Determined by Optical Coherence Tomography Angiography. Am. J. Ophthalmol..

[16] Drexler W., Fujimoto J.G. (2008). State-of-the-Art Retinal Optical Coherence Tomography. Prog. Retin. Eye Res..

[17] Lin A., Fang D., Li C., Cheung C.Y., Chen H. (2020). Improved Automated Foveal Avascular Zone Measurement in Cirrus Optical Coherence Tomography Angiography Using the Level Sets Macro. Transl. Vis. Sci. Technol..

[18] Zhang J., Tang F.Y., Cheung C., Chen X., Chen H. (2021). Different Effect of Media Opacity on Automated and Manual Measurement of Foveal Avascular Zone of Optical Coherence Tomography Angiographies. Br. J. Ophthalmol..

[19] Ishii H., Shoji T., Yoshikawa Y., Kanno J., Ibuki H., Shinoda K. (2019). Automated Measurement of the Foveal Avascular Zone in Swept-Source Optical Coherence Tomography Angiography Images. Transl. Vis. Sci. Technol..

[20] Shoji T., Ishii H., Kanno J., Sasaki T., Yoshikawa Y., Ibuki H., Shinoda K. (2021). Distance Between the Center of the FAZ Measured Automatically and the Highest Foveal Bulge Using OCT-Angiography in Elderly Healthy Eyes. Sci. Rep..

[21] Philippakis E., Legrand M., El Sanharawi M., Erginay A., Couturier A., Tadayoni R. (2018). Measurement of Full-Thickness Macular Hole Size Using En Face Optical Coherence Tomography. Eye.

[22] Gass J.D.M. (1995). Reappraisal of Biomicroscopic Classification of Stages of Development of a Macular Hole. Am. J. Ophthalmol..

[23] Chen Y., Nasrulloh A.V., Wilson I., Geenen C., Habib M., Obara B., Steel D.H.W. (2020). Macular Hole Morphology and Measurement Using an Automated Three-Dimensional Image Segmentation Algorithm. BMJ Open Ophthalmol..

[24] Liefers B., Venhuizen F.G., Schreur V., van Ginneken B., Hoyng C., Fauser S., Theelen T., Sánchez C.I. (2017). Automatic Detection of the Foveal Center in Optical Coherence Tomography. Biomed. Opt. Express.

