# Correction: Sasaki et al. Automatic Determination of the Center of Macular Hole Using Optical Coherence Tomography En Face Images. *J. Clin. Med.* 2022, *11*, 3167

**DOI:** 10.3390/jcm12010392

**Published:** 2023-01-03

**Authors:** Takanori Sasaki, Takuhei Shoji, Junji Kanno, Hirokazu Ishii, Yuji Yoshikawa, Hisashi Ibuki, Kei Shinoda

**Affiliations:** Department of Ophthalmology, Saitama Medical University, Saitama 350-0495, Japan

## Text Correction

There was an error in the original publication [1]. **IRB 19079.03**.

A correction has been made to **2. Materials and Methods**, **2.1. Study Population**:

**IRB 19079.02**

There was an error in the original publication [1]. **November 2019**.

A correction has been made to **2. Materials and Methods**, **2.1. Study Population**:

**September 2019**

## Back Matter Correction

There was an error in the original publication [1]. **IRB 19079.03**.

A correction has been made to **Institutional Review Board Statement**:

**IRB 19079.02**

The authors apologize for any inconvenience caused and state that the scientific conclusions are unaffected. The original publication has also been updated.
